# Motor pool selectivity of neuromuscular degeneration in type I spinal muscular atrophy is conserved between human and mouse

**DOI:** 10.1093/hmg/ddae190

**Published:** 2024-12-18

**Authors:** Justin C Lee, Wendy K Chung, David J Pisapia, Christopher E Henderson

**Affiliations:** Center for Motor Neuron Biology and Disease, Columbia University Medical Center, 630 W. 168th St., New York, NY 10032, United States; Department of Neurosurgery, Baylor College of Medicine, 7200 Cambridge St. Ste. 9B, Houston, TX 77030, United States; Department of Pediatrics, Boston Children’s Hospital, Harvard Medical School, 300 Longwood Ave., Boston, MA 02115, United States; Department of Pathology, Weill Cornell Medical Center, 520 E. 70th St., New York, NY 10021, United States; Center for Motor Neuron Biology and Disease, Columbia University Medical Center, 630 W. 168th St., New York, NY 10032, United States; Alltrna, Inc., 325 Vassar St. Ste. 2A, Cambridge, MA 02142, United States

**Keywords:** spinal muscular atrophy, neurodegeneration, neuromuscular, motor unit, motor neuron

## Abstract

Spinal muscular atrophy (SMA) is caused by low levels of the survival motor neuron (SMN) protein. Even though SMN is ubiquitously expressed, the disease selectively affects motor neurons, leading to progressive muscle weakness. Even among motor neurons, certain motor units appear more clinically resistant to SMA. To quantitatively survey selective resistance, we studied extensive neuromuscular autopsies of Type I SMA patients and age-matched controls. We found highly divergent degrees of degeneration of neighboring motor units, even within individual cranial nerves or a single anatomical area such as the neck. Examination of a Type I SMA patient maintained on life support for 17 years found that most muscles were atrophied, but the diaphragm was strikingly preserved. Nevertheless, some resistant human muscles with preserved morphology displayed nearly complete conversion to slow Type I myofibers. Remarkably, a similar pattern of selective resistance was observed in the SMNΔ7 mouse model. Overall, differential motor unit vulnerability in human Type I SMA suggests the existence of potent, motor unit-specific disease modifiers. Mechanisms that confer selective resistance to SMA may represent therapeutic targets independent of the SMN protein, particularly in patients with neuromuscular weakness refractory to current treatments.

## Graphical Abstract

**Graphical Abstract:**
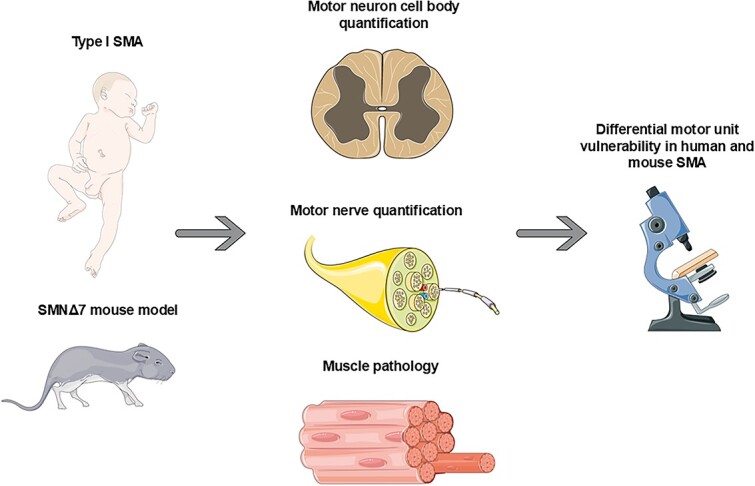

## Introduction

Selective degeneration of a subset of neurons in response to loss or dysfunction of a ubiquitously expressed gene is a hallmark of many neurodegenerative diseases, including Alzheimer’s disease, Parkinson’s disease and amyotrophic lateral sclerosis (ALS) [1–3]. Understanding the molecular basis of selective neurodegeneration may provide insights into disease mechanisms and help identify disease-modifying therapeutic targets. Identification of such modifiers requires analysis not only of the neuronal class affected, but also of the specific subsets of neurons in that class which are most vulnerable [4]. However, our knowledge of the vulnerability of specific subpopulations in patients with neurodegenerative disease remains limited.

To elucidate the molecular determinants of selective vulnerability, use of animal models is necessary. Therefore, for each disease it is critical to rigorously compare the patterns of selective vulnerability between the mouse genetic model and the human disease. The results have been encouraging in the case of ALS, which differentially affects specific motor pools, discrete groups of motor neurons in the spinal cord that control contraction of individual muscles. The most commonly studied mouse model of ALS overexpresses the mutant superoxide dismutase 1 (*SOD1*) gene, the cause of ~ 2% of all human cases of ALS, at high levels [5]. Nevertheless, the mouse models precisely reproduce the selective resistance of motor neurons innervating the extraocular muscles (EOMs) and pelvic sphincters—and the selective vulnerability of fast spinal motor neurons—observed in all sporadic ALS patients [4, 6–9].

Proximal spinal muscular atrophy (SMA), the most frequent genetic cause of infant death prior to the development of disease-modifying treatments, is caused by autosomal recessive deletions or mutations in the ubiquitously expressed survival motor neuron 1 (*SMN1*) gene [10]. Partial compensation by increasing copy number of the paralog survival motor neuron 2 (*SMN2*) leads to progressively milder phenotypes. The carrier frequency of *SMN1* mutation is 1/54, leading to a historical pan-ethnic incidence of approximately 1/11000 live births [11, 12]. However, the increasingly widespread use of reproductive carrier screening is lowering this incidence. Low levels of the survival motor neuron protein (SMN) cause widespread motor neuron loss, leading to death from respiratory failure by two years of age in the most affected Type I patients (OMIM 253300) in the absence of treatment [13–16].

The development of FDA-approved disease-modifying treatments to increase the functional level of SMN protein represents a major advancement, producing significant functional improvements and milestone achievements in many patients [17–26]. Indeed, these improvements in survival and motor function are producing new disease phenotypes, such as Type I SMA patients that can sit but may develop scoliosis not typically seen in less affected Type II SMA patients [27]. Despite this remarkable development, many treated patients still exhibit neuromuscular weakness, including some Type I patients with failure to obtain independent sitting or even persistent ventilator dependence or death and some later-onset SMA patients who fail to exhibit motor improvements [18, 21, 22, 28, 29]. All current SMA treatments under clinical development focus on increasing levels of SMN. Thus, the characterization of differential motor neuron vulnerability, which may lead to the identification of therapeutic pathways independent of SMN, remains highly relevant.

The degree to which different motor neuron subpopulations are clinically affected varies widely. Despite generalized flaccid paralysis with areflexia, Type I SMA patients retain normal eye movements and generally have preserved facial expression and external sphincter continence [30–32]. Additionally, relative sparing of the diaphragm in conjunction with severe functional impairment of the intercostal muscles often produces a ‘bell-shaped chest’ and paradoxical breathing that is virtually pathognomonic for this disorder, as noted by Werdnig as early as 1891 [33]. Reflecting this clinical picture, previous neuropathological studies in Type I SMA report widespread loss of spinal and cranial motor neurons but selective preservation of the oculomotor nuclei (III, IV, and VI), the phrenic nucleus, and Onuf’s nucleus [30, 34–38]. However, detailed characterization of motor neuron vulnerability among motor pools has not been reported.

Differential vulnerability has been more rigorously characterized in SMA model mice, which as in human patients exhibit neuromuscular degeneration that is dependent on levels of SMN protein. The SMNΔ7 mouse, which survives until approximately P13 [39], is the most widely used and best-characterized mouse model of SMA. Ling et al. (2012) documented differential vulnerability in many axial and appendicular muscles in the SMNΔ7 mouse but the degree to which this is correlated with denervation in patients was not determined [40].

Here we report the pathological examination of neuromuscular autopsies in Type I SMA patients and an age-matched control patient and found that the large, fast α-motor neurons that innervate Type II myofibers are more affected than are the slow motor units. In addition, independent of this subtype specificity, in over 80 motor units examined we found remarkable variation in pathology at the level of muscle, nerve, and motor neuron cell bodies. There was a striking concordance between the pattern observed in human patients and selective motor pool vulnerability in the SMNΔ7 mouse. Intriguingly, even in morphologically unremarkable SMA-resistant muscles from patients, dramatic fiber-type conversion to Type I myofibers occurred, suggesting ongoing denervation. Nevertheless, the diaphragm in a Type I SMA patient who died at 17 years exhibited remarkable preservation. Together, our findings indicate that pool-specific determinants of SMA vulnerability lead to marked differences in disease severity that are conserved across species, suggesting that mechanistic conclusions drawn from the study of this aspect of the mouse model may be highly relevant for therapeutic strategies in patients.

## Results

### Variations in motor unit pathology in type I SMA patients

We studied ten autopsies of children with Type I SMA ranging from 4 to 8 months of age and an aged-matched control from a patient who died at 7 months from congenital diaphragmatic hernia (detailed clinical history in Supplementary Table 1), with the aim of determining the range of pathology present. None of the patients in this study received disease-modifying treatment, since all succumbed to SMA prior to the advent of these therapies.

**Figure 1:**
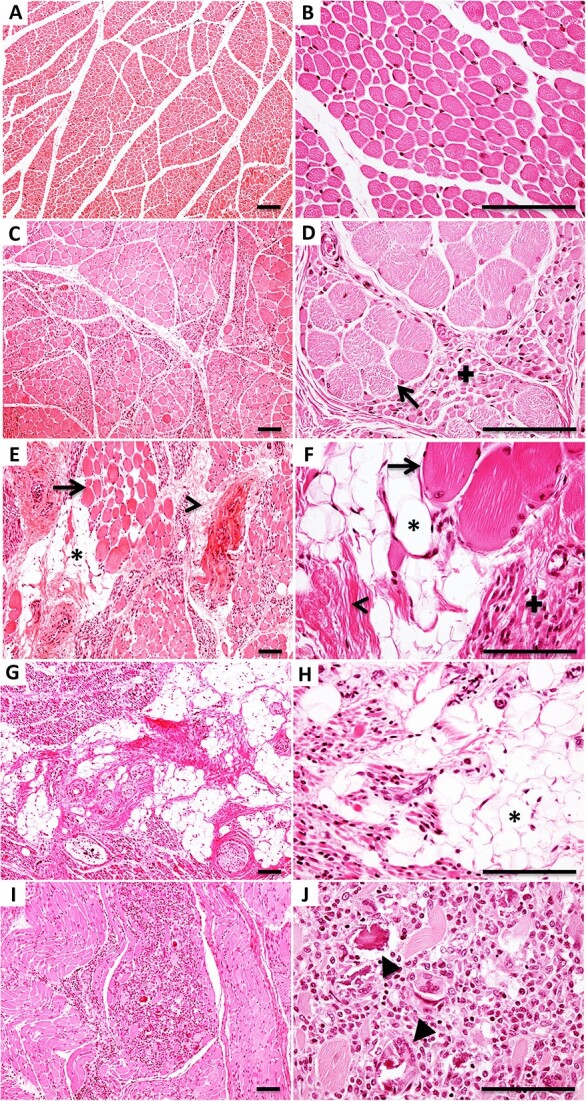
Pathological features used to classify human muscles with different degrees of denervation. (A and B) psoas muscle from control patient (A) with high-magnification field (HMF) in (B). Control muscles have relatively little variation in myofiber diameter and regular architecture in all samples examined. (C and D) moderately affected psoas from type I SMA patient (C) with HMF in (D). Moderately affected muscles displayed varying degrees of denervation atrophy (plus sign in D) and compensatory hypertrophy (arrow in D) of remaining fibers. (E and F) severely affected tibialis anterior from type I SMA patient (E) with HMF in (F). Severely affected muscles displayed increased connective tissue composed of collagen fibrils (thin arrowheads in E,F) and adipocytes (asterisks in E,F), in addition to denervation atrophy (plus sign in F) and compensatory hypertrophy (arrows in E,F). (G and H) soleus from type I SMA patient (G) with HMF in (H). This was among the most severely affected muscles, with large amounts of fibro-fatty connective tissue (H, asterisk). (I and J) deltoid from type I SMA patient (I) with HMF in (J). Focal inflammatory infiltrates and multinucleated giant cell digestion of myofibers are present (J, filled arrowheads). H&E staining. Scale bar, 100 μm.

**Table 1:** Classification of muscles according to degree of denervation pathology in type I SMA patients.

				**Rostrocaudal position of motor pool**									
**Muscle**													
**Axial (cranial)**	Brainstem	C1-C2	C3-C4	C5-C6	C7-C8	T1-T4	T5-T8	T9-T12	L1-L2	L3-L4	L5-S1	S2-S3	S4-S5
Temporalis													
Extraocular													
Mylohyoid													
Anterior digastric													
SCM													
Trapezius													
**Axial**													
Sternothyroid													
Thyrohyoid													
Sternohyoid													
Paraspinals													
Internal intercostals													
External intercostals													
Diaphragm													
Rectus abdominus													
EAS													
**Appendicular**													
Deltoid													
Psoas													
Vastus lateralis													
Tibialis anterior													
Soleus													
Gastrocnemius													
Lumbricals (foot)													
**Classification of pathology:**													
Spared													
Moderately affected													
Severely affected													
Most severe													

Pathology was observed in at least six H&E-stained cross-sections through the longitudinal extent of the muscle. Four classes of severity were defined as follows: 1. Spared (green): no significant pathology by H&E staining 2. Moderately affected (yellow): widespread variation in myofiber diameter from denervation atrophy and compensatory hypertrophy 3. Severely affected (red): marked myofiber variation with increased connective tissue and fatty infiltrates 4. Most severely affected (violet): largely replaced by fibro-fatty infiltrates with denervation atrophy in most remaining fibers. SCM = sternocleidomastoid; EAS = external anal sphincter.

We first established a standardized classification of muscle pathology, which we used as an indicator of motor unit vulnerability. All observations were based on blinded examination of at least six representative hematoxylin and eosin (H&E)-stained transverse sections throughout the longitudinal extent of each muscle. Control muscles displayed normal architecture and relatively uniform myofiber diameter, with no apparent pathology in any muscle examined (Fig. 1A and B). Among muscles from SMA patients, we defined four classes of severity of denervation pathology: spared, moderately affected, severely affected, and most severely affected (Table 1). ‘Spared’ muscles were indistinguishable by H&E staining from controls. ‘Moderately affected’ SMA muscles showed marked variation in myofiber diameter: denervated myofibers were severely atrophied while myofibers that remained innervated or were re-innervated displayed hypertrophy, likely in response to increased workload (Fig. 1C and D). ‘Severely affected’ muscles exhibited even greater variation in myofiber diameter and increases in endomysial connective tissue and fatty infiltrates (Fig. 1E and F). Lastly, the ‘most severely affected’ muscles contained large amounts of fibro-fatty replacement tissue (Fig. 1G and H). In rare severely affected muscles, we also observed focal endomysial inflammatory infiltrates and digestion of myofibers by multinucleated giant cells of monocyte–macrophage lineage as confirmed with immunohistochemistry (IHC) for CD68 (Fig. 1I and J; Suppl. Fig. 1). The degree of denervation pathology in each muscle was reproducible between individual Type I SMA patients (Suppl. Fig. 2). For some muscles, such as the external anal sphincter (EAS) and the cricopharyngeus, for which control samples were not available in this study, it was not possible to classify the pathology. Even in healthy controls [41–43], these muscles exhibit features that are considered pathological in other muscles, such as heterogeneity in myofiber diameter, increased endomysial connective tissue (up to 40%), and alterations in myofiber-type distribution (Suppl. Fig. 3D–I).

Degeneration of motor units in SMA was also apparent from examination of motor axons and cell bodies. L5 ventral roots showed a marked loss of myelinated motor axons (Fig. 4F) as compared to controls (Fig. 4A). Correspondingly, examination of approximately 35 spinal cord segments from nine Type I SMA patients revealed a significant depletion of motor neuron cell bodies in the ventral horn of all tissues examined (Fig. 3A and B).

**Figure 2:**
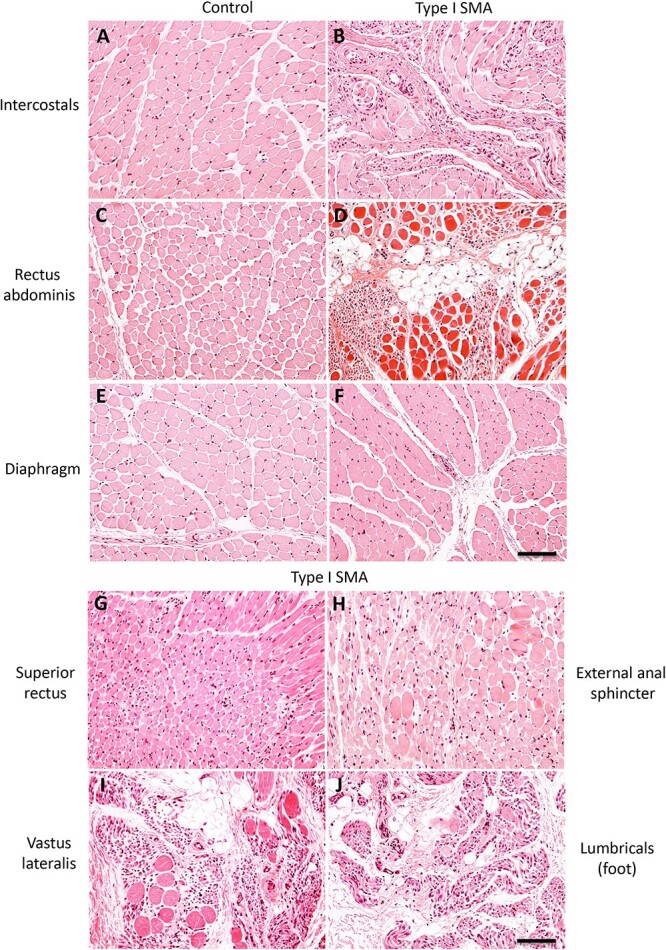
Denervation pathology in different human muscles matches the clinical phenotype. (A–F) muscles of respiration from a type I SMA patient and a control patient. Type I SMA intercostals (B) and rectus abdominis (D) exhibit moderate denervation pathology, which is absent from corresponding control muscles (A and C). Type I SMA diaphragm (F) is unremarkable compared to control (E). (G) the superior rectus from a type I SMA patient is spared. (H) the external anal sphincter from type I SMA patient had morphology consistent with sparing. (I) the vastus lateralis from type I SMA patient is among the most severely affected muscles. (J) Foot lumbricals from type I SMA patient exhibits severe denervation pathology. H&E staining. Scale bar, 100 μm.

### Motor unit pathology reflects differential clinical vulnerability

To analyze the selectivity of disease resistance, we first focused on motor units for which there is clinical evidence of differential vulnerability. Given the ‘bell-shaped chest’ observed clinically, we compared the pathology of the diaphragm, intercostals, and rectus abdominis by H&E staining. The intercostal and rectus abdominis muscles were moderately affected in Type I SMA and showed marked denervation atrophy and variation in myofiber diameter, with some areas displaying increased connective tissue and fibro-fatty infiltrates (Fig. 2A–D). In contrast, the diaphragm exhibited only minor alterations in myofiber diameter and slightly increased connective tissue, but was largely spared (Fig. 2E and F).

**Figure 3:**
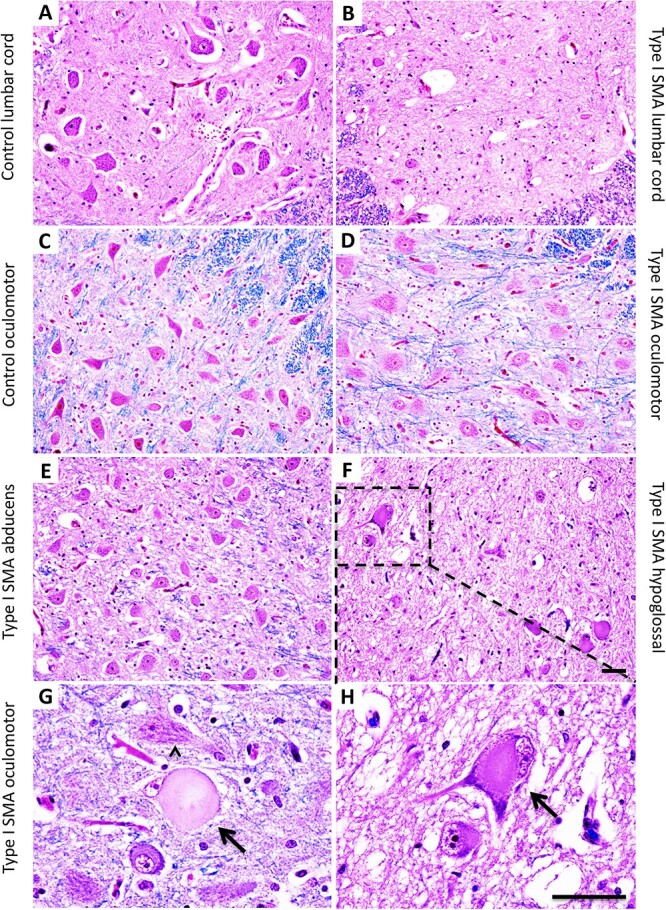
Varying degrees of SMA vulnerability at the level of human motor neuron cell bodies. (A and B) ventral horn of lumbar spinal cord in a type I SMA patient (B) was largely depleted of motor neuron cell bodies compared to control (A). (C–E, and G) extraocular motor nuclei from type I SMA and control patients. The cell bodies in type I SMA oculomotor (D) (high-magnification field (HMF) in G) and abducens (E) were largely intact, with healthy features such as euchromatic nuclei with prominent nucleoli and Nissl bodies evenly distributed throughout the cytoplasm (arrowhead in G) that were observed in the control oculomotor nucleus (C). In type I SMA rare oculomotor neurons (< 0.5%) exhibited degeneration (arrow in G), with chromatolysis, ballooning, and peripheral displacement of nuclei. (F and H) hypoglossal nucleus in a type I SMA patient. The hypoglossal nucleus (F) with HMF in (H) was also highly affected, with reduced motor neurons and pathological features, such as central chromatolysis and peripheral or dendritic displacement of Nissl substance and nuclei (arrow in H), in most remaining cell bodies. H&E-luxol fast blue staining. Scale bar, 50 μm.

**Figure 4:**
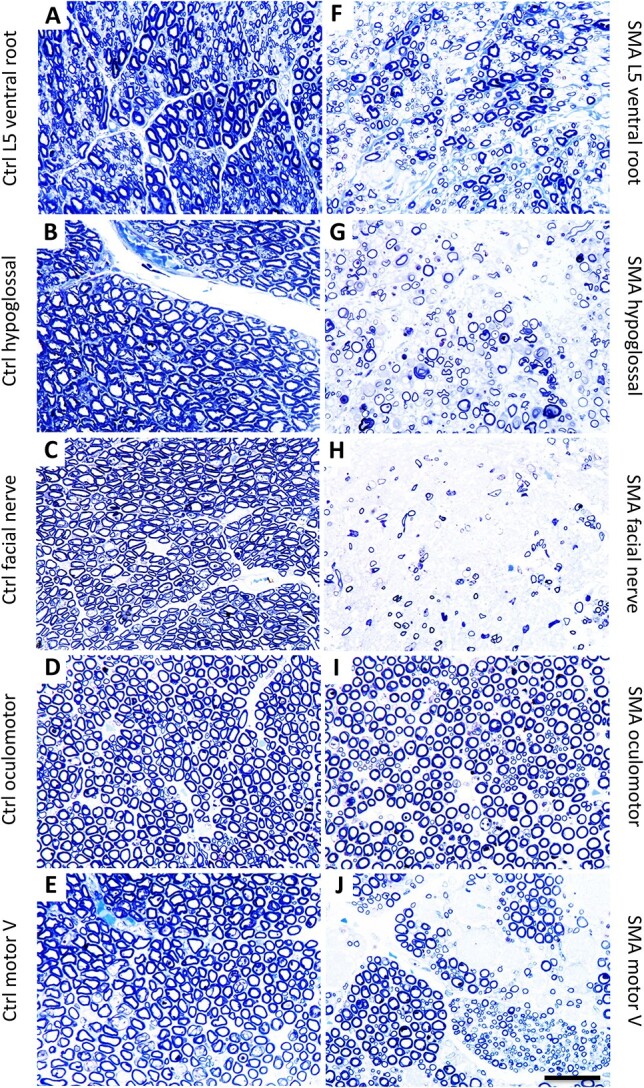
Differential motor unit vulnerability at the level of peripheral motor axons in human SMA patients. (A–E) control spinal and cranial motor nerves exhibited dense, compact arrays of myelinated axons. (F–J) type I SMA spinal and cranial motor nerves had varying degrees of axon loss. (A and F) L5 ventral root in type I SMA (F) exhibited diffuse loss of myelinated axons, compared to control patient (A). (B and G) hypoglossal nerve in type I SMA patient (G) had marked axon loss compared to control (B). (C and H) facial nerve in type I SMA (H) was largely depleted of myelinated axons compared to control nerve (C). (D and I) the oculomotor nerve in type I SMA (I) was largely intact compared to control (D), with a relatively small reduction in density of myelinated fibers. (E and J) the motor V nerve in type I SMA (J) had some fascicles that were depleted of myelinated axons compared to control (E), while others appeared to contain a full complement of motor axons. Toluidine blue-stained semi-thin sections. Scale bar 40 μm.

**Figure 5:**
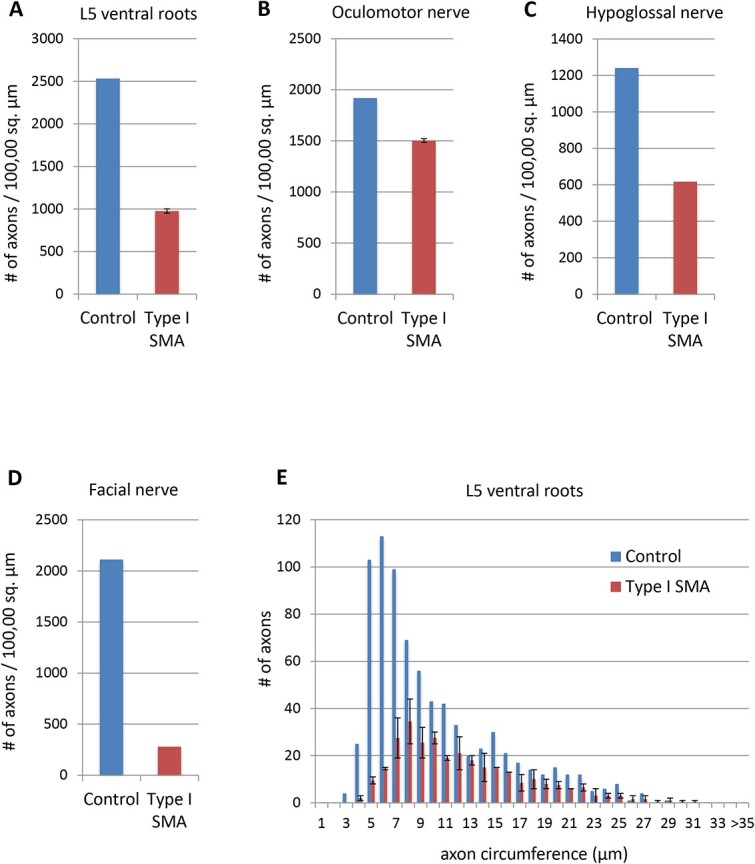
Density of myelinated motor axons in ventral roots and cranial nerves type I SMA and control patients. (A and E) density and distribution of axons in L5 ventral root in type I SMA and control. There is a 62% reduction in density of myelinated axons in type I SMA (A). Distribution of L5 axon circumference (E) reveals widespread loss, especially pronounced among smaller axons. (B) Oculomotor nerve in type I SMA compared to control reveals modest 22% reduction in axon density in SMA. (C) Hypoglossal nerve in type I SMA compared to control reveals marked 50% reduction in density in SMA. (D) Facial nerve in type I SMA compared to control reveals large 87% reduction in density in SMA. Values are means, error bars are mean ± SEM, *n* = 2.

Patients with SMA retain extraocular motor function, even at late stages of the disease. Reflecting their clinical resistance, EOMs such as the superior rectus (Fig. 2G) were remarkably spared. Moreover, both oculomotor and abducens nuclei showed remarkable preservation of motor neuron cell bodies in SMA patients, except for rare degenerating cells (Fig. 3C–E, and G). To quantify this difference, we used axon profiles in the corresponding peripheral motor nerves. In the control patient, all nerves showed dense, compact arrays of myelinated and non-myelinated axons (Fig. 4A–E). Examination of the L5 spinal nerve in Type I SMA revealed a large 62% depletion of myelinated axons (Figs. 4F, 5A and E). In contrast, most myelinated profiles in the oculomotor nerve remained intact with only 22% of axons lost (Figs. 4I, 5B). To more precisely define the pattern of motor neuron loss, we plotted the distribution of motor axon size. In L5 ventral roots, axons were lost across the size spectrum, including large-diameter axons likely corresponding to α-motor neurons and small-diameter γ-motor fibers (Fig. 5E). By contrast, in the oculomotor nerve, the small number of axons lost was largely restricted to the intermediate size category (Suppl. Fig. 4A). Motor neuron preservation or loss was highly similar between SMA patients, as reflected by the low variation in total axon loss and distribution of axon size, suggesting a highly stereotyped pattern of vulnerability (Fig. 5, Suppl. Fig. 4). In SMA patients, involvement of other cranial nerves is frequently manifest through tongue fasciculations and bulbar dysfunction [32, 44]. We observed a 50% loss of myelinated axons in the hypoglossal nerve, selectively affecting the large axons (Figs. 4B and G, 5C, Suppl. Fig. 4B). We correspondingly found a large depletion of motor neuron cell bodies in the hypoglossal nucleus, with most remaining cells exhibiting marked chromatolysis with peripheral displacement of Nissl substance and nuclei (Fig. 3F and H).

Overall, the clinical preservation of eye movement compared to dysfunction of other cranial motor nerves in SMA patients is matched by clear differential vulnerability of the corresponding motor units, particularly the large α-motor neurons. However, such parallels may not be perfect: whereas Type I SMA patients generally retain facial expression [31, 45, 46], we detected motor axon loss in the facial nerve that was disproportionately severe compared to the clinical findings (Figs. 4C and H, 5D, Suppl. Fig. 4C). Access to facial muscles was not permitted at autopsy so it was not possible to determine whether muscle denervation was similarly pronounced.

### Differential vulnerability of muscles innervated by a single nerve

One potential explanation for the differences in disease resistance observed thus far was that the different motor pools and nuclei are situated at different rostrocaudal levels. To determine whether differences in vulnerability also exist between motor pools situated at similar levels, we studied two nuclei—the spinal accessory (XI) and the motor trigeminal (V)—each of which innervates multiple muscles.

**Figure 6:**
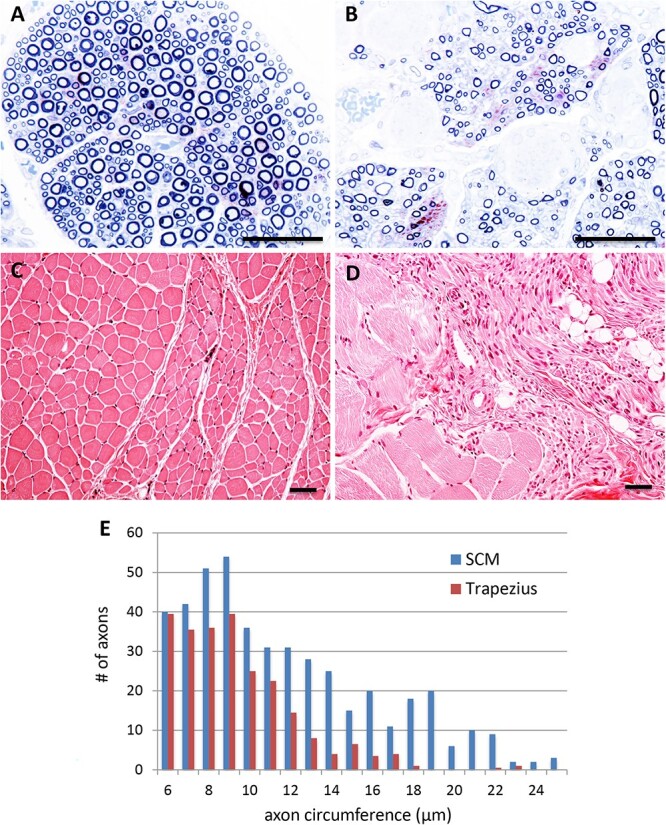
Differential vulnerability within the spinal accessory (XI) nerve of a type I SMA patient. (A and B) motor axons innervating the sternocleidomastoid (SCM) (A) are remarkably preserved compared to those innervating the trapezius (B). (C-D) SCM muscle (C) and trapezius muscle (D) are relatively spared and vulnerable, respectively, reflect the selective axonal loss in (A andB). (E) Distribution of axon circumference reveals preferential loss of the large α-motor neurons in the trapezius as compared to the SCM. A&B: Toluidine blue semi-thin sections; C&D: H&E staining. Scale bars, 50 μm.

The spinal accessory (XI) nerve innervates two muscle targets: the sternocleidomastoid (SCM) and the trapezius (it provides the sole innervation for the descending part of the trapezius [47]). Within the XI nerve, the fascicles corresponding to each muscle are readily distinguished morphologically. Strikingly, in the XI nerve from an SMA patient, the SCM fascicle contained large numbers of both large and small myelinated axons, whereas the trapezius fascicle was significantly depleted of large myelinated axons (Fig. 6A, B,and E). Comparison to corresponding fascicles in the control patient demonstrated a selective loss of the large alpha motor neurons that innervate the trapezius in SMA (Suppl. Fig. 4). Muscle pathology closely matched this observation: although the SCM muscle displayed increased variation in myofiber diameter, indicating some denervation pathology, it was mostly spared compared to the descending trapezius, which was among the severely affected muscles in this study (Fig. 6C and D).

**Figure 7:**
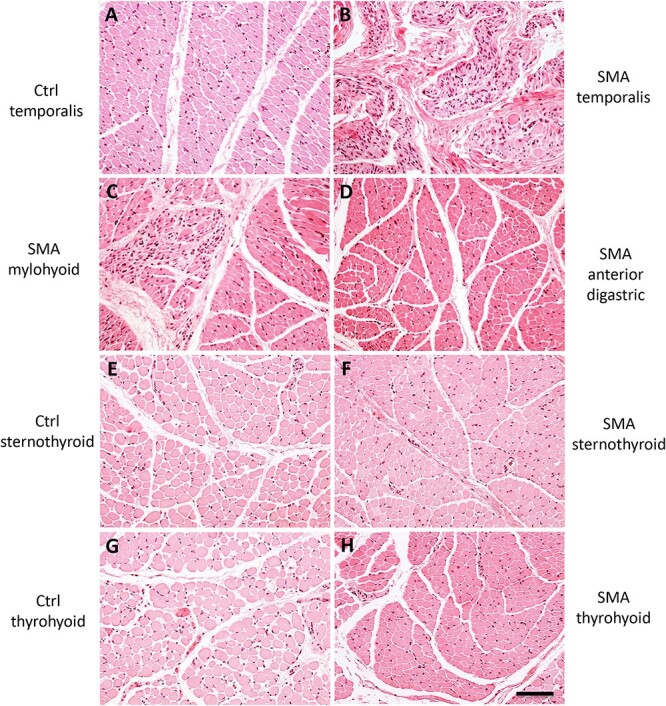
Remarkable preservation of select ventral neck and infrahyoid muscles in type I SMA patients. (A andB) temporalis in type I SMA (B) was severely affected compared to control muscle (A). (C) the mylohyoid in type I SMA was affected to an intermediate degree. (D) the anterior belly of the digastric in type I SMA was devoid of denervation pathology. (E and H) Infrahyoid muscles in type I SMA and control. The sternothyroid (F) and thyrohyoid (H) in type I SMA were remarkably spared compared to corresponding control muscles (E and G) in all areas examined. H&E staining. Scale bar, 100 μm.

The motor V nerve innervates multiple muscles involved in chewing and swallowing. In SMA patients, it exhibited a pattern of degeneration comparable to that for the spinal accessory nerve: some fascicles appeared to be relatively preserved, whereas others showed profound axonal loss (Fig. 4E and J). In this nerve it is not possible to distinguish the fascicles that correspond to individual muscles on morphological grounds alone. We therefore analyzed target muscles of the motor V in SMA patients and the one muscle, the temporalis, available from the control patient. The temporalis was severely affected in SMA (Fig. 7 A and B). In marked contrast, the anterior belly of the digastric (Fig. 7D) appeared almost normal. The mylohyoid was affected, but only moderately (Fig. 7C). This suggested that, as with XI, fascicles corresponding to different muscles within the single motor V nerve exhibit marked differential vulnerability to low levels of the SMN protein.

**Figure 8:**
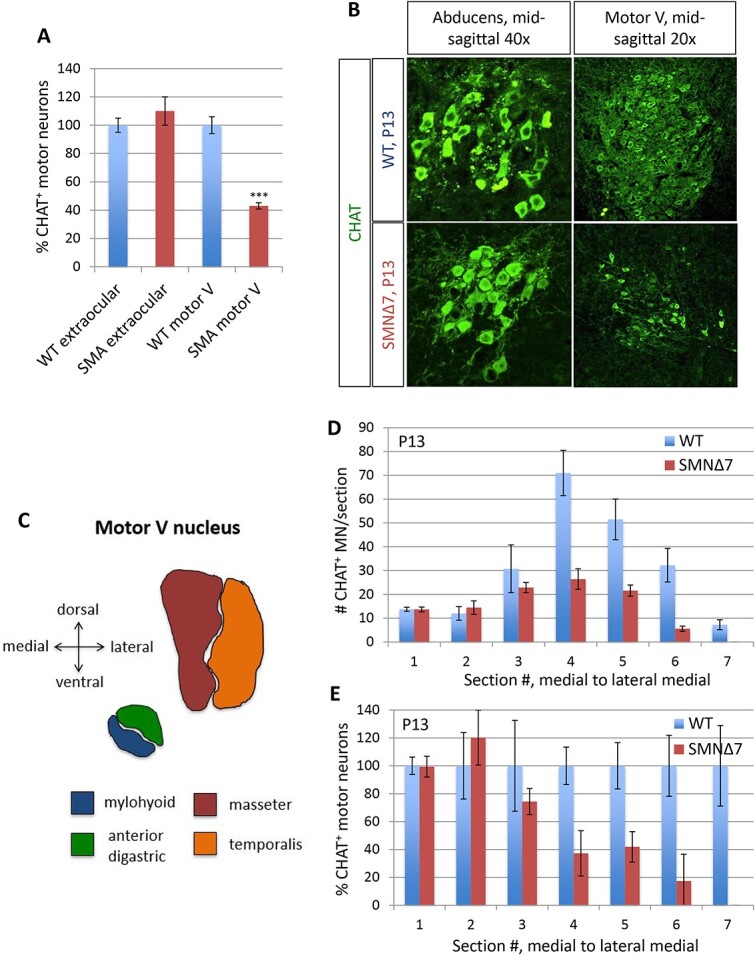
Preferential loss of the lateral motor V nucleus in SMNΔ7 mice. (A and B) the motor V, but not the extraocular motor nuclei, exhibited a large reduction in ChAT-positive motor neuron cell bodies by P13 end-stage SMNΔ7 mice (B). Quantification (A) confirmed a 57% reduction in motor V, but no reduction in the extraocular motor nuclei. (C andE) motor neuron loss in motor V plotted from medial to lateral. There was a preferential loss of the lateral motor V (C) as quantified by motor neuron number (D) and percentage lost (E) in SMNΔ7 compared to wild type mice. Values are means ± SEM, *n* = 4–6 per genotype.

Since not all target muscles of the motor V from SMA patients were accessible to us due to lack of consent, we turned to the SMNΔ7 mouse, the most frequently used mouse model of SMA, to complement this part of the study. Strikingly, whereas loss of motor neuron cell bodies at end-stage in SMNΔ7 mice is relatively modest in most regions of the spinal cord [39, 48–50], we observed a 58% loss in the motor V nucleus in P13 end-stage mice (Fig. 8A and B). The oculomotor neurons in SMNΔ7 mice, in contrast, were fully preserved (Fig. 8A and B). This raised the possibility that, as in human patients, some motor V neurons are selectively vulnerable. To analyze motor neuron loss at a more refined level, we quantified motor V motor neurons across a medial-to-lateral axis. Due to the relative spatial distribution of the motor pools within the nucleus, this allowed us to count successively the motor neurons that innervate the anterior digastric or mylohyoid, then the temporalis, and finally the masseter muscle (Fig. 8C). In P13 end-stage SMNΔ7 mice, we found a dramatic loss of motor neurons that innervate the temporalis or masseter, but no significant loss of the medial motor neurons that innervate the mylohyoid or anterior digastric (Fig. 8D).

**Figure 9:**
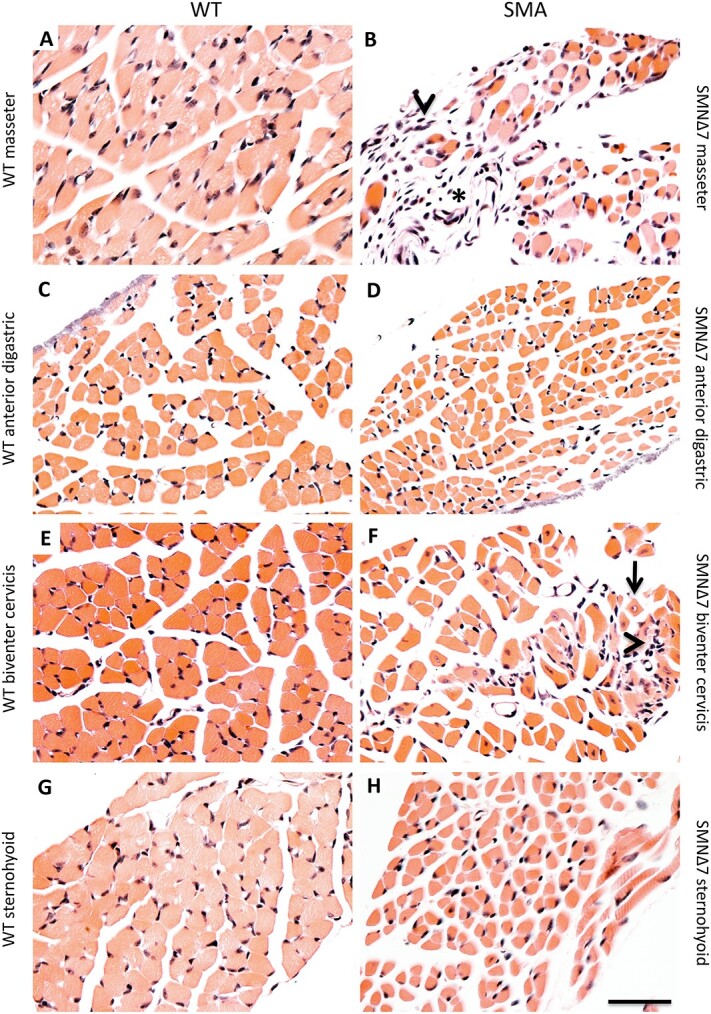
The SMNΔ7 mouse recapitulates differential vulnerability seen in human SMA at the level of individual muscles. (A and B) the masseter in SMNΔ7 mice (B) exhibited marked variations in myofiber diameter, increased connective tissue (asterisk in B), and groups of nuclei that represent putative denervated myofibers (arrowhead in B). These features were never present in control masseter from wild-type littermates (A). (C-D) the anterior belly of the digastric in SMNΔ7 mice (D) had relatively uniform myofiber diameter and lacked features of denervation pathology. Myofibers were smaller than in corresponding control muscle (C). (E andF) the biventer cervicis in SMNΔ7 mice (F) exhibited the features of denervation pathology and a marked increase in central nuclei (arrow in F) that was not present in the control muscle (E). (G and H) the sternohyoid muscle (H) was spared from denervation pathology, although also had smaller myofibers compared to control muscle (G). N = 3 per muscle/genotype. H&E staining. Scale bar, 40 μm.

We therefore examined the pathology of the corresponding muscles. Studies to date have quantified denervation in SMNΔ7 mice by measuring co-localization of IHC for presynaptic motor terminals and motor endplates [40]. However, in our hands, results of staining in neonatal muscle were variable due to unequal distribution of denervation within individual muscles and the ubiquity of partially innervated neuromuscular junctions that required a qualitative assessment of presynaptic terminal overlap with motor endplates; muscle examination by H&E staining more reliably detected denervation pathology. Moreover, it provided a direct parallel with our studies of human tissue. Control masseter showed regular, closely packed fibers (Fig. 9A), whereas the masseter from SMNΔ7 mice exhibited disordered architecture, variation in myofiber diameter, increased endomysial connective tissue, and clumps of nuclei that likely represent denervated myofibers (Fig. 9B). In contrast, the anterior digastric from SMNΔ7 mice retained normal architecture and relatively uniform myofiber diameter (Fig. 9D), although individual fibers were smaller, reflecting the substantially reduced overall size of SMA mice, which weigh 68% less than control littermates at end-stage (data not shown).

The SMNΔ7 mouse therefore remarkably recapitulates the differential motor pool vulnerability within the motor V nucleus that we observed in human Type I SMA patients. Moreover, the mouse and human data show that, within a single motor nucleus, there can co-exist widely divergent degrees of vulnerability to SMA.

### Stereotyped differential vulnerability of neck motor units

Characteristic features of untreated Type I SMA include the failure to gain neck control and frequent bulbar dysfunction. Thus, the relative sparing of the anterior digastric and the SCM—both neck muscles—was unexpected. We therefore further characterized the vulnerability of neck muscles responsible for maintaining head position and airway patency. Given the striking parallels between the SMNΔ7 mouse and SMA patients in the specificity of motor V pathology, we studied the mouse and human diseases in parallel.

Examination of H&E-stained neck muscles in the SMNΔ7 mouse revealed that dorsal neck muscles that contribute to head stability and movement, such as the biventer cervicis, were severely affected (Fig. 9E and F), with clear evidence of denervation pathology, including disordered architecture and an increase in central nuclei (Fig. 9F). In contrast, the sternohyoid, which is a ventral infrahyoid neck muscle that depresses the hyoid and larynx during swallowing, was spared (Fig. 9G and H).

The sparing of a major ventral neck muscle as compared to dorsal neck muscles in the mouse was striking. We therefore examined the corresponding ventral muscles of the neck available to us from Type I SMA patients to determine if this was a previously unappreciated aspect of the human disease. All infrahyoid muscles examined—the sternohyoid, sternothyroid, and thyrohyoid—were remarkably spared, and indeed were virtually indistinguishable from control muscles by H&E staining in all areas examined (Fig. 7E–H). Thus, there is a remarkable degree of conservation between mouse and human in the exquisitely selective pattern of vulnerability to low SMN in both the trigeminal nucleus and a diverse array of cervical musculature.

### Onset of neuromuscular pathology preceding morphological degeneration

Thus far, our studies determined muscle pathology by morphological examination of classical H&E staining, supported by IHC examination of neuromuscular junctions in select muscles. This approach allowed us to determine differential vulnerability, but only detected overt denervation pathology. Since some SMA-resistant muscles were virtually indistinguishable from controls by H&E, we asked whether they might have undergone more subtle changes. We turned to muscle fiber-type grouping, which reflects the spatial reorganization of individual surviving motor units that re-innervate neighboring myofibers following their denervation.

**Figure 10:**
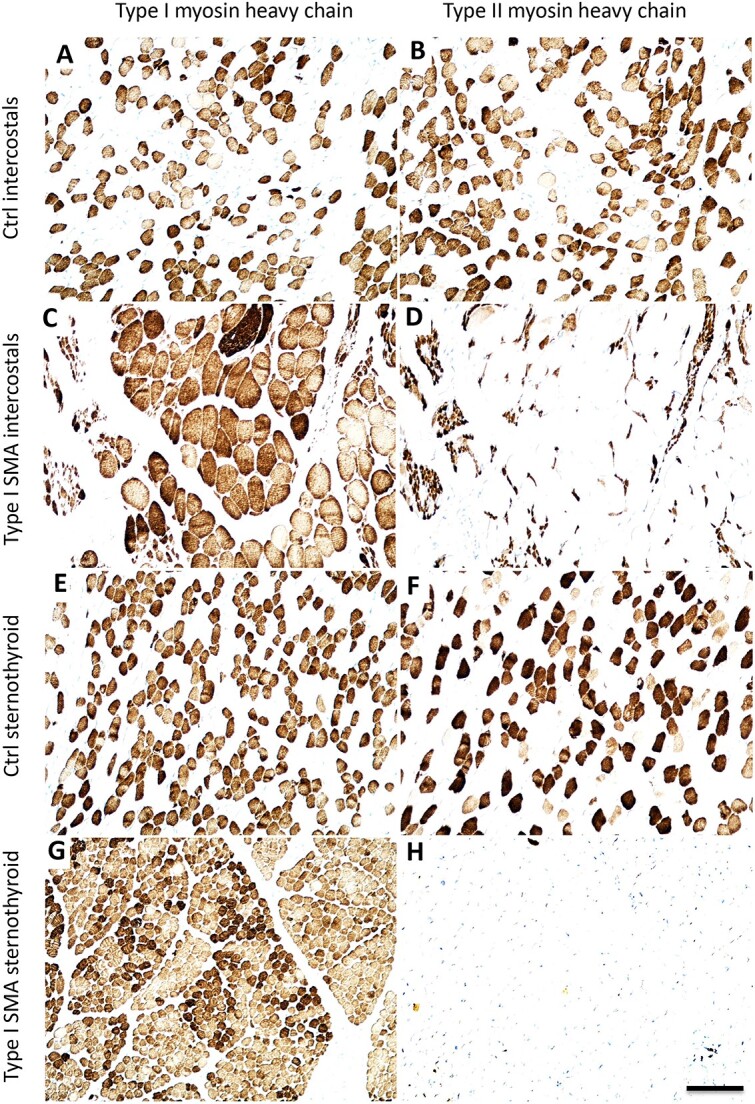
Conversion from type II (fast) to type I (slow) myofibers in type I SMA patients, even in morphologically normal muscles. Myosin heavy chain immunohistochemistry: Type I (left panels), type II (right panels) on serial sections. (A–D) intercostal muscles from type I SMA and control patient. Control patient exhibited a normal checkerboard pattern of type I (A) and type II (B) myofibers. Type I SMA patients had massive fiber type grouping, myofiber conversion, and compensatory hypertrophy of type I myofibers (C). Most of the remaining type II myofibers (D) exhibited severe denervation atrophy. (E–H) Sternothyroid muscle from type I SMA and control patient. The sternothyroid in type I SMA exhibited almost complete conversion from type II (H) to type I (G) myofibers. Corresponding control muscle had a normal complement of type I (E) and type II (F) myofibers. Scale bar, 100 μm.

Using antibodies specific to Type I (slow) and Type II (fast) myosin, we first analyzed the highly vulnerable intercostal muscles from a Type I SMA patient. Instead of the normal checkerboard pattern of Type I and Type II myofibers observed in control muscle (Fig. 10A and B), we observed massive conversion to Type I myofibers in the SMA muscle as previously reported (Fig. 10C). Most of the remaining Type II myofibers exhibited pronounced denervation atrophy (Fig. 10D).

We next performed the same analysis on muscles that have highly preserved function and minimal changes by H&E staining. The superior rectus exhibited a predominance of Type II myofibers, with Type I myofibers distributed uniformly throughout the global and orbital layers (Suppl. Fig. 3A–C). The EAS, which is also reportedly spared in SMA [38], exhibited a predominance of Type I myofibers, with Type II myofibers distributed uniformly throughout the muscle (Suppl. Fig. 3D–F). Although these two muscles have differing fiber-type compositions to meet their divergent functional demands, the uniform fiber distribution throughout these muscles suggests an absence of motor unit reorganization.

In contrast, the infrahyoid muscles, despite having morphology that was virtually indistinguishable from control muscle by H&E, exhibited a dramatic conversion to Type I myofibers. The sternothyroid, for example, had almost no remaining Type II fibers (Fig. 10G and H). This suggests that by disease end-stage in human patients, even some of the most SMA-resistant muscles may exhibit chronic denervation, with a marked preference for denervation of Type II myofibers, but that successful reinnervation by slow motor axons is sufficient to completely delay the appearance of gross morphological changes.

### Preservation of SMA-resistant motor units in prolonged type I human SMA

Clinically, in Type I SMA distal muscles are initially spared relative to proximal muscles and patients may retain movement in their wrists, hands, and feet [30, 32, 51]. By late disease stages, however, even distal limb muscles are paralyzed and show denervation pathology as severe as that of proximal limb muscles (e.g. foot lumbricals; Fig. 2J). Nevertheless, some motor units such as the diaphragm and infrahyoid muscles are morphologically spared even in late stages of disease. Since Type I SMA patients typically succumb to respiratory failure within the first two years of life [13–16], measuring the absolute degree of preservation of these motor units is usually not possible.

**Figure 11:**
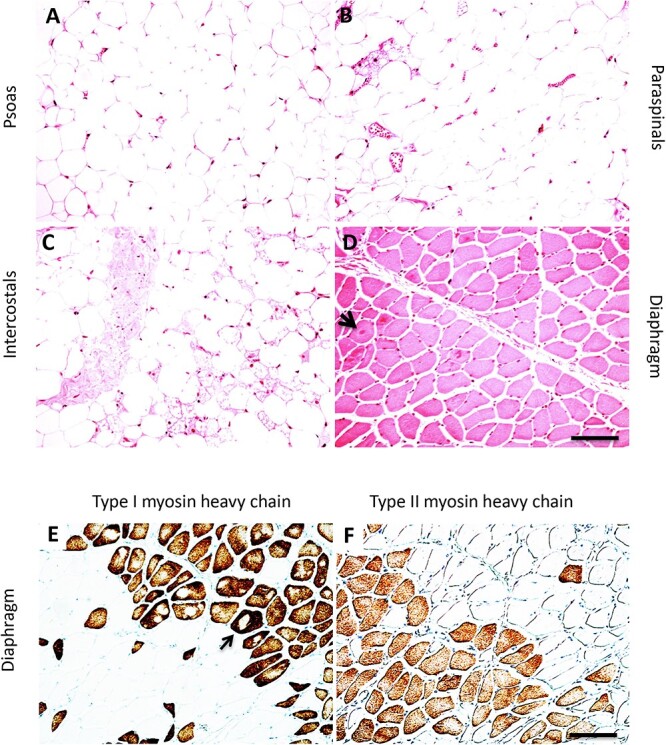
Remarkable preservation of the diaphragm in a type I SMA patient at 17 years of age. (A–C) all affected muscles examined, including the psoas (A), paraspinals (B), and intercostals (C), were replaced almost entirely with fat cells and connective tissue with very few remaining myofibers. (D) the diaphragm appeared relatively normal and did not exhibit features of denervation pathology, with the exception of putative target or targetoid myofibers (arrow in D). (E and F) the diaphragm exhibited greater preservation of type II myofibers (F), which tended to be grouped, presumably from collateral sprouting. Many type I myofibers (E) in this patient exhibited putative target or targetoid fibers in type I myofibers (arrow in E). This was not frequently observed in the type II myofibers of this patient (F), or in the diaphragm of patients with a shorter clinical course (Suppl. Fig. 5). H&E staining (A–D). Myosin heavy chain immunohistochemistry: Type I (E), type II (F). Serial sections. Scale bars 100 μm.

We therefore examined muscles from a Type I SMA patient who was maintained on respiratory support for 17 years, significantly longer than the 7-month average lifespan of the Type I SMA patients in our study. Affected muscles, including the intercostal muscles, displayed dramatic pathology: myofibers were virtually entirely replaced by fat and connective tissue (Fig. 11A–C). The diaphragm, however, was remarkably spared (Fig. 11D). Interestingly, in contrast to the resistant ventral neck muscles described above, the diaphragm exhibited a substantial number of Type II myofibers, generally grouped as a result of collateral sprouting (Fig. 11E and F). We also noted the presence of putative target or targetoid fibers (Fig. 11D, arrow), which are thought to represent an early sign of denervation [52, 53]. This pathology was largely restricted to slow Type I myofibers (Fig. 11E, arrow)). In contrast, three other Type I SMA patients who were examined, all of whom died within the first few months of life, showed substantially greater conversion of the diaphragm to Type I myofibers (Suppl. Fig. 5) and did not show prominent targetoid fibers (Fig. 2B; Suppl. Fig. 5). These results demonstrate that, at least in some patients, the diaphragm exhibits remarkable preservation for many years and, unlike other muscles, fails to switch completely to expression of slow myosin. The clinical history shows that this patient also retained normal oculomotor function through his entire disease course, indicating that long-term resistance extends to other SMA-resistant motor units.

## Discussion

We report a remarkably wide range in vulnerability of different motor pools in Type I SMA, even though—prior to disease-modifying therapies—these children often survived only a few months. Future such studies may be impossible, given the widespread adoption of FDA-approved therapies that markedly alter the clinical outcomes in Type I SMA. This characterization provides a rational basis for cardinal aspects of the clinical phenotype and opens a path to identification of disease modifiers representing potent therapeutic targets independent of the SMN protein. Our finding that the SMNΔ7 mouse closely mimics selective motor pool resistance in patients provides rationale for its use as a preclinical model for neuromuscular denervation and suggests that mechanistic conclusions drawn from its study may be relevant in human patients. Lastly, the existence of early molecular changes in muscles that remain histologically normal suggests that some degree of degeneration occurs in even the most SMA-resistant motor pools. These pools may exhibit a rate of degeneration that is below the threshold for overt morphological changes or may better compensate for motor unit loss with a higher capacity for collateral sprouting. Moreover, the prolonged resistance of some motor pools with long survival times demonstrates that SMA-resistance may be nearly absolute.

### Widespread motor neuron loss in type I SMA

The limited degree of motor neuron loss reported to date at end-stage in the SMNΔ7 mouse (likely reflecting the mouse-specific cardiac phenotype) has led to diminished emphasis on the contribution of motor neuron cell death to the clinical phenotype. Nevertheless, our extensive examination of motor neuron loss in Type I SMA patients revealed a large depletion of motor neuron cell bodies in the ventral horn of the spinal cord and select cranial motor nuclei (Fig. 3). We found a corresponding 50–87% loss of myelinated axons in spinal and cranial motor nerves, in agreement with classical pathological studies (Figs. 4 and 5). Thus, even in patients who survived only 4 months, motor neuron degeneration was a major pathological finding at autopsy.

These findings are consistent with a widespread and rapid motor neuron loss in Type I SMA patient and clinical trials have underscored the need to intervene before this degeneration and corresponding clinical weakness. The phase III ENDEAR study utilizing nusinersen, an antisense oligonucleotide that increases the proportion of full-length SMN protein, found that symptomatic Type I SMA patients were more likely to exhibit a motor-milestone response and had a prolonged time to death or needing permanent ventilation [21]. However, only 51% of symptomatic patients had a motor-milestone response and only 8% achieved independent sitting. In contrast, the NURTURE study examining the effects of nusinersen in pre-symptomatic infants under 6 weeks of age with 2–3 copies of *SMN2* found that 100% of patients acquired the ability to sit independently and nearly 90% achieved independent walking [19, 20]. Similar results were obtained in the SPR1NT trial treating pre-symptomatic Type I SMA infants with onasemnogene abeparvovec, an adeno-associated viral serotype 9 (AAV9) vector utilized to deliver copies of wild-type *SMN*. All infants treated at less than 6 weeks of age sat independently; 9/14 patients with two *SMN2* copies walked at 18 months of age and 14/15 with three *SMN2* copies walked independently at 24 months of age [54, 55].

Moreover, in the SMNΔ7 mouse we demonstrate extensive motor neuron deficits in previously unstudied structures such as the motor V nucleus (Fig. 8) and certain segments of the lumbar medial motor column (not shown). Since the ~ 60% loss of trigeminal motor neurons involves largely masseter and temporalis neurons, the fractional loss in these populations may be closer to 90% (Fig. 8E).

This does not preclude the involvement of other cell types, such as proprioceptive sensory neurons, interneurons, or glia in the pathogenic process [50, 56–62]. Indeed, the current consensus is that to generate the full extent of SMA pathology it is necessary to lower SMN in diverse cell types [56]. However, to the degree to which we can determine, motor pools or nuclei in which motor neurons survive show preserved function clinically. This serves as a reminder of the potential clinical significance of SMN-independent neuroprotective treatments to retard motor neuron degeneration [56].

### Graduated degrees of motor unit resistance to SMA

Despite pronounced motor neuron loss overall, we found considerable differences in the degree to which individual motor pools and their corresponding muscles are affected, even at autopsy. Our examination of over 80 muscles from Type I SMA patients revealed a marked heterogeneity (Table 1). Many muscles were mildly to moderately affected, with variations in myofiber diameter resulting from concurrent denervation atrophy and compensatory hypertrophy of the remaining innervated myofibers. These moderately affected muscles are similar to those reported in the literature [30, 32, 45, 63, 64]. However, we also identified motor units that were more severely affected than previously thought, with varying degrees of fibro-fatty infiltration (Fig. 1; Table 1); muscles such as the vastus lateralis and soleus were essentially atrophic by end-stage, even in patients who were not maintained on life support. This occurs despite the human soleus containing ~ 80% Type I slow-twitch fibers, which we confirmed to be more resistant on average than Type II fast motor units. Thus, the influence of motor pool-specific modifiers may in some cases overcome motor neuron subtype-dependent properties [9].

Type I SMA patients previously did not survive beyond two years of age, making it hard to determine the absolute degree of resistance of a given motor pool. It was therefore significant that a representative SMA-resistant muscle, the diaphragm, was preserved in a Type I SMA patient at 17 years, whereas all other muscles examined were virtually entirely replaced with fat cells. The presence in the diaphragm of this patient of target or targetoid fibers, which are thought to represent an early, reversible sign of denervation and have been observed in Type III, but not Type I SMA [52, 53, 63], suggests that some degree of chronic neuromuscular denervation may continue for many years in surviving SMA patients.

Another interpretation of these findings is that once a given motor unit has overcome a critical stage in the progression of the disease, it can survive for a considerable period provided the patient remains alive. Studies in the SMNΔ7 mouse indeed suggest that there is a high requirement for SMN in the early postnatal period, with reduction of SMN in the adult mouse producing virtually no gross motor, morphological or electrophysiological phenotype [65–67]. The clinical trial data discussed above suggests a similar early critical period exists in patients and may explain the lack of diaphragm pathology progression at later stages [68]. Restoration of the SMN protein above a critical threshold may halt disease progression and preclude the phase of chronic neuromuscular denervation observed in the diaphragm of the untreated Type I SMA patient that we examined.

The differential response of different motor pools to therapies that increase SMN protein remains an outstanding question. Analysis of differentially vulnerable motor units in the SMNΔ7 mouse following postnatal rescue of SMN levels using an antisense oligonucleotide confirmed that the loss of axons and neuromuscular junction endplates depends on the vulnerability of the underlying motor unit [69]. Despite widespread axonal sprouting in moderately affected motor units in response to SMN treatment, recovery was incomplete; severely affected motor units exhibited marked pathology even following early treatment at P1 [69]. Muscle-specific functional assessments in patients is difficult, especially in infants that are unable to cooperate with complex motor tasks. However, the presence of paradoxical inspiratory inward motion of the pulmonary ribcage in the setting of weak intercostal muscles with a relatively preserved diaphragm in Type I SMA patients allows for the assessment of intercostal motor unit response to treatment. LoMauro and colleagues found that SMA subtype IC (clinical onset between 3 and 6 months) but not subtypes IA (onset within two weeks of life) or IB (onset within 3 months) treated with nusinersen had a significant improvement in paradoxical ribcage motion with a corresponding reduction in the daily hours of mechanical ventilation required [70]. These findings support the notion that treatment prior to a critical threshold in neuromuscular degeneration is required for clinical improvements in function, which may differ by motor unit subtype.

### Spatial and functional correlates of motor unit vulnerability

Given the wide array of motor units examined in this study, we assessed whether any motor unit characteristics might be associated with vulnerability. We considered two: neuroanatomical position and firing rate. Position along the rostrocaudal axis did not predict vulnerability, as we found that resistant and vulnerable populations co-exist at all levels. One striking example was provided by the spinal accessory nerve (XI), which in the past has been classified as strongly affected in SMA [30]. Nevertheless, since the spinal accessory nucleus contains two motor pools—SCM and trapezius—we analyzed them separately. Their axons are readily distinguished morphologically by fascicle size; this is not possible in spinal ventral roots, which contain axons from as many as ten motor pools at a single rostrocaudal segment [71]. Although both motor units were affected, we found only modest pathology in the SCM (Fig. 6, Suppl. Fig. 4D and E), but marked degeneration of the trapezius, even though the cell bodies of the two pools are positioned closely in the spinal cord and have similar initial axon trajectories. A comparable example from the trigeminal motor nucleus is discussed below.

Normal activity levels also vary greatly between different motor pools. Intriguingly, all the SMA-resistant motor pools we identified are among the most highly active of somatic motor units. Oculomotor neurons have a tonic discharge rate of 100 spikes per second at the primary position and exhibit high frequency bursts of up to 600 spikes per second during ballistic movements, an order of magnitude higher than most spinal motor neurons [72]. The phrenic motor neurons fire in a continuous rhythmic pattern, as cessation of this activity is incompatible with life [73]. EAS motor units fire tonically to maintain continence and exhibit substantial increases in firing during any activity that increases intra-abdominal pressure, even speaking [74, 75]. A quantitative electromyographic study comparing the suprahyoid muscle (which we find to be SMA-resistant) to the temporalis, masseter and tibialis anterior muscles (which we show to be vulnerable) found that the suprahyoid muscle was much more active than the others, even during sleep [76]. A final correlation is provided by the fact that most intercostal muscles, which are strongly affected in SMA, are not active during normal respiration or sleep [77]. Overall, our study supports the hypothesis that high levels of activity of a given motor pool may correlate with SMA resistance. This would also help to explain the relative resistance of slow over fast spinal motor neurons, since the latter only fire in infrequent bursts. In the future, it will be interesting to determine the SMA phenotype of other highly active motor pools, such as the scalenes and the parasternal intercostals, which are primary muscles of inspiration [78]. Further characterization of neuromuscular pathology will also form the basis for determining the molecular mechanisms underlying selective motor unit vulnerability in human and mouse SMA. Prior studies have successfully utilized this strategy to identify Matrix metalloproteinase-9 as a marker for vulnerable motor units and a potential therapeutic target in ALS [4].

### Subclinical changes in resistant motor units detected by histopathology

The young age and fragility of Type I SMA patients prohibits most functional exploration. This means that moderate dysfunction of some muscle groups may remain clinically undetected. Moreover, among motor units that are still completely functional, some may be completely resistant whereas others may be about to reach the limits of functional compensation. Motor units that are on the borderline of degeneration may represent critical targets for future therapies. We therefore used quantitative morphological and immunohistochemical criteria to identify them.

The first example was the oculomotor nuclei, whose function is strikingly preserved in Type I SMA. Indeed, in our study motor neuron cell bodies of the oculomotor and abducens nuclei appeared largely intact (Fig. 3C-E). Nevertheless, quantification of CN III in Type I patients who died at 7 months detected a ~ 20% loss of motor axons. Close pathological examination of the oculomotor nucleus confirmed a small number of actively degenerating motor neurons (Fig. 3G). This confirms some degree of motor unit loss in SMA-resistant motor units, but the rate of motor unit loss is below the threshold for clinically evident oculomotor dysfunction. Furthermore, the EOMs from Type I SMA patients did not exhibit any features of denervation pathology. However, the EOMs are known to exhibit unique structural and functional properties and may exhibit a relatively mild response to denervation that would preclude detection of pathology [72, 79–82]. Therefore, our examination of cell bodies, motor axons, and clinical history indicates that the extraocular motor units are highly SMA-resistant but may have been on the threshold of degeneration at end-stage in these patients.

A second example of subclinical changes emerged when we analyzed fiber-type grouping using antibodies to fast and slow myosin. Surprisingly, we found that even SMA-resistant muscles with minimal to no morphological change observable by H&E exhibited dramatic myofiber rearrangements. These results indicate that the fast α-motor neurons that innervate Type II myofibers are selectively and severely affected, even in some of the most SMA-resistant motor pools. The increase in the number of Type I fibers likely reflects collateral sprouting by the remaining slow motor neurons analogous to that underlying fiber-type grouping in milder forms of SMA [32]. By extension, these data suggest that resistance of a given motor pool is determined by the properties of the remaining slow α-motor neurons, perhaps by their pool-specific capacity for collateral sprouting. Interestingly, a progressive switch from fast to slow motor units has also been reported in the SOD1 mouse model of ALS [83, 84].

A final example of mismatch between the clinical and morphological phenotypes is harder to explain. We found a > 85% loss of motor axons in the facial nerve of Type I SMA patients, although their control of facial expression is generally retained. Some clinicians have noted ‘bland’ facial expression, attributed to relative facial weakness [30]. However, another study found no clinically detectable facial weakness in ~ 80% of SMA patients [31]. Since we could not access facial muscles for autopsy, the amount of denervation pathology and the apparent discrepancy between motor unit loss and clinical motor function in this case remains to be fully elucidated.

### The SMNΔ7 mouse recapitulates vulnerability in human SMA

Although the SMNΔ7 mouse reproduces the generalized reduction in SMN levels that characterizes human patients, significant divergence from the classical SMA phenotype, such as a pronounced cardiac phenotype and a relatively modest degree of motor neuron cell death have been emphasized [39, 48–50], raising the question as to its reliability as a model for the study of the human disease. To determine whether we could gain insights into human SMA from studies in mouse models, we compared the selectivity of motor unit degeneration between Type I SMA patients and SMNΔ7 mice.

The motor V nerve in human Type I SMA exhibited marked differences in motor axon loss between fascicles, with some largely depleted of myelinated axons and others retaining a full complement of fibers. H&E staining of the corresponding muscles demonstrated that the laterally located temporalis was severely affected. In contrast, the medially located anterior digastric was virtually unaffected. In P13 SMNΔ7 mice, there was a ~ 60% loss of motor V cell bodies, the most severe reported in the SMNΔ7 mouse to date [50, 85]. In a remarkable parallel to our findings in Type I human SMA, there was a marked preferential loss of the lateral motor neurons that innervate the masseter and the temporalis, but no significant loss of the medial ones that innervate the mylohyoid and the anterior digastric (Fig. 8). H&E staining of the corresponding muscles confirmed this selective vulnerability (Fig. 9). In further support of our findings, a study used MRI to examine bulbar muscles in Type II SMA patients and found that the laterally positioned temporalis and masseter were severely affected with mild to severe atrophy and fatty infiltration, while the medial geniohyoid and digastric were less affected [86].

The parallel between mouse and human models is further reinforced by our observation that the ventral neck muscles in both species (where available) were highly preserved as compared to dorsal neck muscles. In the SMNΔ7 mouse a major ventral neck muscle, the sternohyoid, was spared relative to the dorsal biventer cervicis. In Type I SMA patients, the ventrally located SCM, anterior digastric, and infrahyoid muscles were highly preserved by H&E staining, despite profound clinical neck weakness in these patients.

However, there is some discordance in pathology observed in human and mouse SMA. Some muscles that are severely affected in Type I SMA patients, such as the trapezius, deltoid, and lumbricals have been found to remain fully innervated in end-stage SMNΔ7 mice [40]. These differences may be attributed to the relatively short life span of this mouse model, with certain motor pools requiring a longer disease course to exhibit denervation. On the other hand, more severe mouse models such as the *SMN2^+/+^; Smn^−/−^* mouse exhibit approximately 50% neuromuscular denervation in the intercostals (with a corresponding preservation of the diaphragm) as early as E18.5, which is greater than the ~ 25% intercostal denervation found at P12–14 in the SMNΔ7 mouse [40, 87]. These findings suggest that denervation pathology may induced with a sufficiently severe SMA mouse model, although these severe models are compromised by early postnatal death. Despite these differences, there is an overall striking parallel in selective vulnerability between human and mouse SMA, even within individual cranial nuclei.

All currently approved therapies for SMA focus on increasing levels of the SMN protein and their benefits have redefined the clinical spectrum of SMA pathology. However, high cost and barriers to access remain and patients who are treated after symptom onset may still exhibit profound neuromuscular weakness [88]. Therefore, it would seem prudent to explore the existence of therapeutic targets independent of SMN. Identifying the mechanisms underlying, for example, the remarkable resistance of the phrenic nucleus represents an alternative strategy to preserve other vulnerable motor units.

## Materials and methods

### Ethics statement

All human tissue was collected with the consent from patients’ guardians and was conducted in accordance with institutional guidelines. Studies were reviewed by the Columbia University Medical Center institutional review board.

### Human tissue dissection and processing

Brain, spinal cord, motor nerves, and muscles were dissected from Type I SMA patients with the shortest postmortem interval possible and immediately drop fixed in 10% formalin. A neuropathologist (D.J.P.) confirmed the identity of all tissues. Tissue was fixed for seven days. Following fixation, brain, spinal cord, and muscles were paraffin-embedded using standard protocols. Central nervous system tissue was stained with combination H&E-luxol fast blue staining. Muscles were stained with H&E. Motor nerves were embedded in plastic using standard protocols and toluidine blue staining was performed on semi-thin sections.

### Classification of muscle pathology

Muscles were divided into at least six longitudinal segments, followed by embedding and staining as described. Pathological classification was performed blinded to SMA status and muscle identity. Classification was based on the criteria listed above. Overall classification was based on close examination of at least six cross-sections and was performed twice for each muscle to ensure reliability. On average, muscle from three SMA patients was examined for each muscle type. When classification was not consistent across trials or across patients for the same muscle type, a third definitive classification was performed.

### Human motor nerve quantification

At least three 60x images were obtained from toluidine blue-stained semi-thin sections of motor nerves. Representative images were evenly spaced across the cross-sectional motor nerve area to ensure unbiased sampling. The inner circumference of each myelin sheath was manually traced with MetaMorph image software. Axon number, density, and size distribution were plotted graphically.

### Human muscle immunohistochemistry

Human muscles were fixed and processed as described above. Immunohistochemistry for MHC-I, MHC-II, CD68, and MPO were performed using automated staining techniques based on the Ventana Ultra platform.

### Animals

Original breeding pairs of SMA mice used in this study (*Smn^+/−^; SMN2^+/+^;SMNΔ7^+/+^*) were purchased from Jackson laboratory. Pups were genotyped using PCR on tail DNA (see Avila et al., 2007 for exact protocol). All SMA mice had the following genotype: *Smn^−/−^;SMN2^+/+^;SMNΔ7^+/+^*. Littermates heterozygous or homozygous for *Smn* were analyzed as wild-type controls.

### Mouse immunohistochemistry

Mice were transcardially perfused with 10 mL/g of 4% paraformaldehyde (PFA) in phosphate-buffered saline (PBS). Brains were dissected and post-fixed overnight in 4% PFA in PBS, followed by a 2–4 h wash with PBS. Tissue was cryoprotected by 48-h immersion in 30% sucrose in 0.1 M PB. Cryopreserved tissue was embedded in OCT on dry ice. Embedded tissue was stored at −80°C until sectioning. Sagittal brain sections were cut on a cryostat at 20 μm.

Cryosections were incubated with ice cold 50% ethanol for 30 minutes, followed by a PBS wash for 3 × 5 minutes at 4°C. Blocking and permeabilization was performed with a one-hour incubation with 4% goat serum/1% Triton-X/PBS at 4°C. Slides were incubated with goat anti-CHAT (Millipore) at 1:125 in 0.4% goat serum/0.1% Triton-X/PBS at 4°C overnight. Slides were washed with PBS 3x10 minutes. Incubation with anti-goat Alexa Fluor 488 (Life Technologies) in 0.4% goat serum/0.1% Triton-X/PBS was performed at room temperature for 2 h. Slides were washed with PBS 3 × 10 min and glass coverslips were mounted using Vectashield.

### Confocal imaging

Imaging was performed with a confocal microscope (Leica SP5). Z stacks of 20 μm sections were obtained at 2 μm intervals with a 40x objective. CHAT^+^ neuron quantification was performed blinded to genotype. Only motor neurons with a visible nucleus were counted.

## Supplementary Material

Supplementary_Figure_ddae190

Supplementary_Table_1_ddae190
