# Supplementary material for: Motor pool selectivity of neuromuscular degeneration in type I spinal muscular atrophy is conserved between human and mouse
Source: Hum Mol Genet. 2024 Dec 18;34(4):347–67. doi: 10.1093/hmg/ddae190 (PMC11811418; doi:10.1093/hmg/ddae190)
Supplement: Supplementary_Figure_ddae190 [file supplementary_figure_ddae190.zip › Lee et al., 2024 - Supplementary legends.docx]

**Supplementary Table 1. Clinical details of Type I SMA patients and control autopsies.**

Patient ID corresponds to the order in which autopsies were processed and analyzed. All SMA cases were confirmed genetically. Type I SMA patients survived from 4-8 months of age, except for one patient who survived for 17 years with respiratory support.

**Supplementary Figure 1. Areas with focal inflammatory infiltrates are evident in some SMA-vulnerable muscles.**

(**A-F**) Serial sections of deltoid muscle from Type I SMA and control patient. SMA deltoid (**B**) exhibited multinucleated giant cells digesting myofibers (arrowhead in B). This was never observed in deltoid from control muscle (**A**). Immunohistochemistry (IHC) against macrosialin with KP1 (**D**) monoclonal antibodies indicated that these giant cells (arrowhead in D) were primarily of monocyte-macrophage lineage. Some neutrophils were also present in SMA deltoid, as visualized with myeloperoxidase (MPO) IHC (**F**). In contrast, macrophages and neutrophils are not observed in control deltoid, evident by lack of KP1 and MPO (**C** and **E**, respectively).

Serial sections. Scale bar, 100 μm.

**Supplementary Figure 2. Highly reproducible degree of denervation pathology in Type I SMA.**

(**A, D**) The psoas of two Type I SMA patients. Psoas is moderately affected with marked denervation atrophy and compensatory hypertrophy of remaining motor units in both patients.

(**B, E**) The soleus of two Type I SMA patients. Soleus is very severely affected with massive fibro-fatty infiltrates and denervation atrophy of virtually all remaining myofibers in both patients.

(**C, F**) The diaphragm of two Type I SMA patients. The diaphragm is largely preserved in both patients, with only mildly increased variation in myofiber diameter.

Patient ID: SMA-1: E; SMA-2: D, F; SMA-3: A-C. H&E staining. Scale bar, 200 μm.

**Supplementary Figure 3. Examination of fiber-type distribution in atypical motor units in Type I SMA patients.**

Only muscle from SMA patients is shown.

(**A-C**) The superior rectus extraocular muscle appeared normal by H&E staining (**A**) and had a uniform distribution of staining for Type I (**B**) and type II (**C**) myosin heavy chain immunohistochemistry (MHC-IHC), despite a Type II myofiber predominance. This distribution was maintained in both the global (larger diameter myofibers in left side) and orbital (smaller myofibers on right side) layers.

(**D-F**) The external anal sphincter (EAS) exhibited variation in myofiber diameter and increased endomysial connective tissue (**D**), which are normal features in this muscle. Type I (**E**) and Type II (**F**) myofibers were distributed uniformly across the muscle, with a Type I fiber predominance.

(**G-I**) The cricopharyngeal sphincter muscle exhibited marked variation in myofiber diameter (**G**), and a predominance of Type II myofibers that appeared to be grouped (**I**) compared to Type I myofibers (**H**).

H&E staining (A, D). MHC-IHC: Type I (B, E), Type II (C, F). Serial sections. Scale bar, 100 μm.

**Supplementary Figure 4. Size distribution of myelinated motor axons in cranial and cervical nerves of Type I SMA and control patients.**

(**A**) Distribution of oculomotor nerve axons in Type I SMA and control. Note preserved size distribution.

(**B**) Hypoglossal nerve in Type I SMA and control, revealing preferential loss of the largest α-motor neurons in Type I SMA.

(**C**) Facial nerve in Type I SMA and control, demonstrating loss across the entire size range in Type I SMA.

(**D, E**) Selective motor unit loss within the spinal accessory nerve (XI) of a Type I SMA patient compared to a control patient. The distribution of myelinated axons in the sternocleidomastoid branch of the XI in a Type I SMA patient was well preserved compared to those in a control patient (**D**). However, the distribution of myelinated axons in the trapezius branch of the XI in a Type I SMA patient compared to the control patient revealed a substantially greater loss of myelinated axons, especially the largest α-motor neurons (**E**).

Values are means, error bars are mean ± SEM, n = 2.

**Supplementary Figure 5. Varying degrees of Type I myofiber conversion in diaphragm of Type I SMA patients.**

(**A-B**) Control patient had a normal checkerboard pattern of Type I (**A**) and Type II (**B**) myofibers.

(**C-H**) Myofiber distribution in Type I SMA patients who died at eight (**C, D**), eight (**E, F**), and five (**G, H**) months of age. All had substantial conversion from Type II (**D, F, H**) to Type I (**C, E, G**) myofibers.

(**I-J**) The Type I SMA patient who remained on life support until 17 years of age had greater preservation of Type II myofibers (**J**), which tended to be grouped, presumably from collateral sprouting. Many Type I myofibers (**I**) in this patient exhibited putative target or targetoid fibers in Type I myofibers (arrow in I). This was not frequently observed in the Type II myofibers of this patient (**H**), or in the patients with a shorter clinical course (**C-H**).

Myosin heavy chain immunohistochemistry: Type I (left panels), Type II (right panels). Serial sections. Scale bar 100 μm.
