# Supplementary figures and images for: Motor pool selectivity of neuromuscular degeneration in type I spinal muscular atrophy is conserved between human and mouse
Source: Hum Mol Genet. 2024 Dec 18;34(4):347–67. doi: 10.1093/hmg/ddae190 (PMC11811418; doi:10.1093/hmg/ddae190)

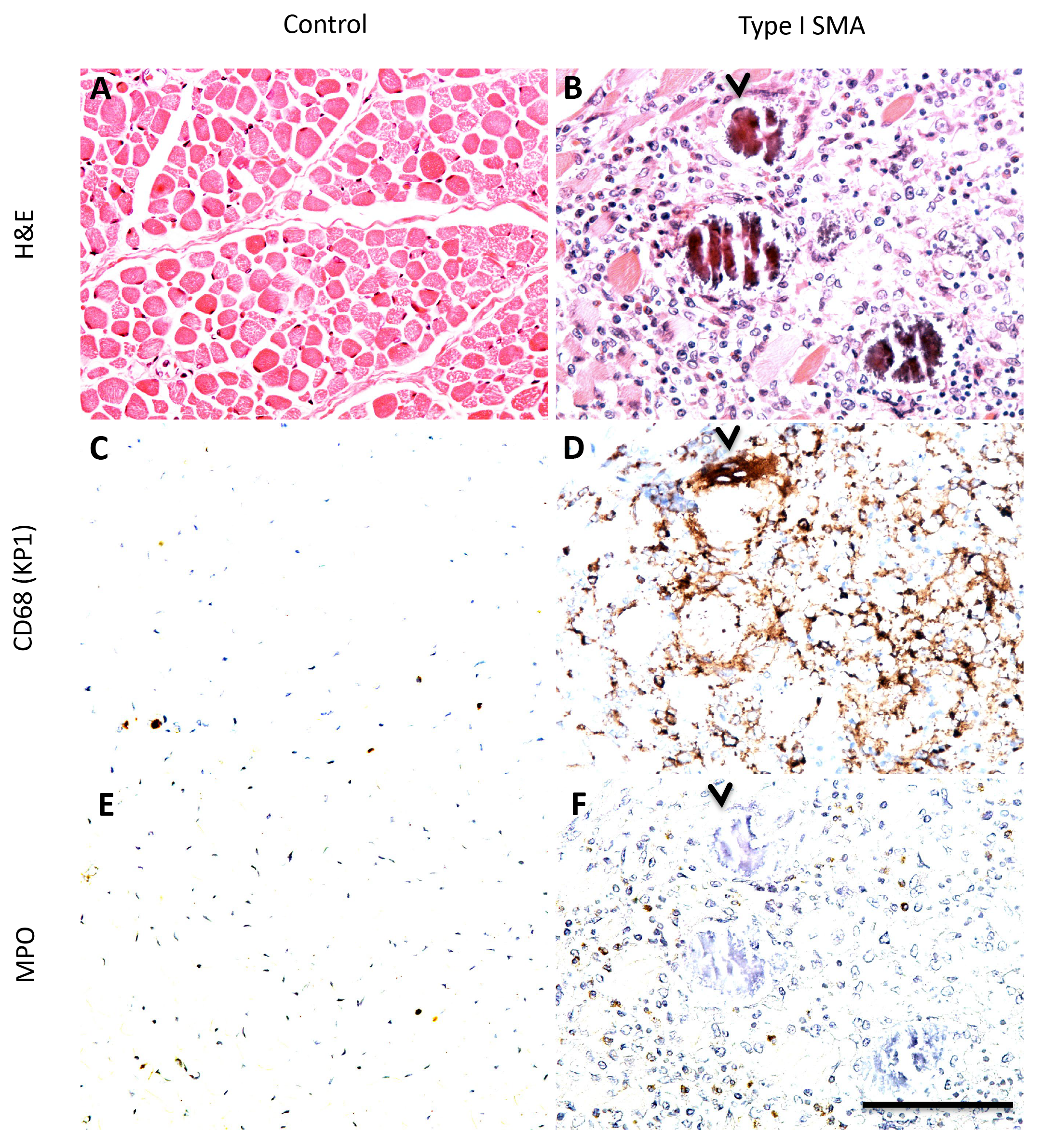

Supplement: Supplementary_Figure_ddae190 [file supplementary_figure_ddae190.zip › Supplementary Figure 1.tif]

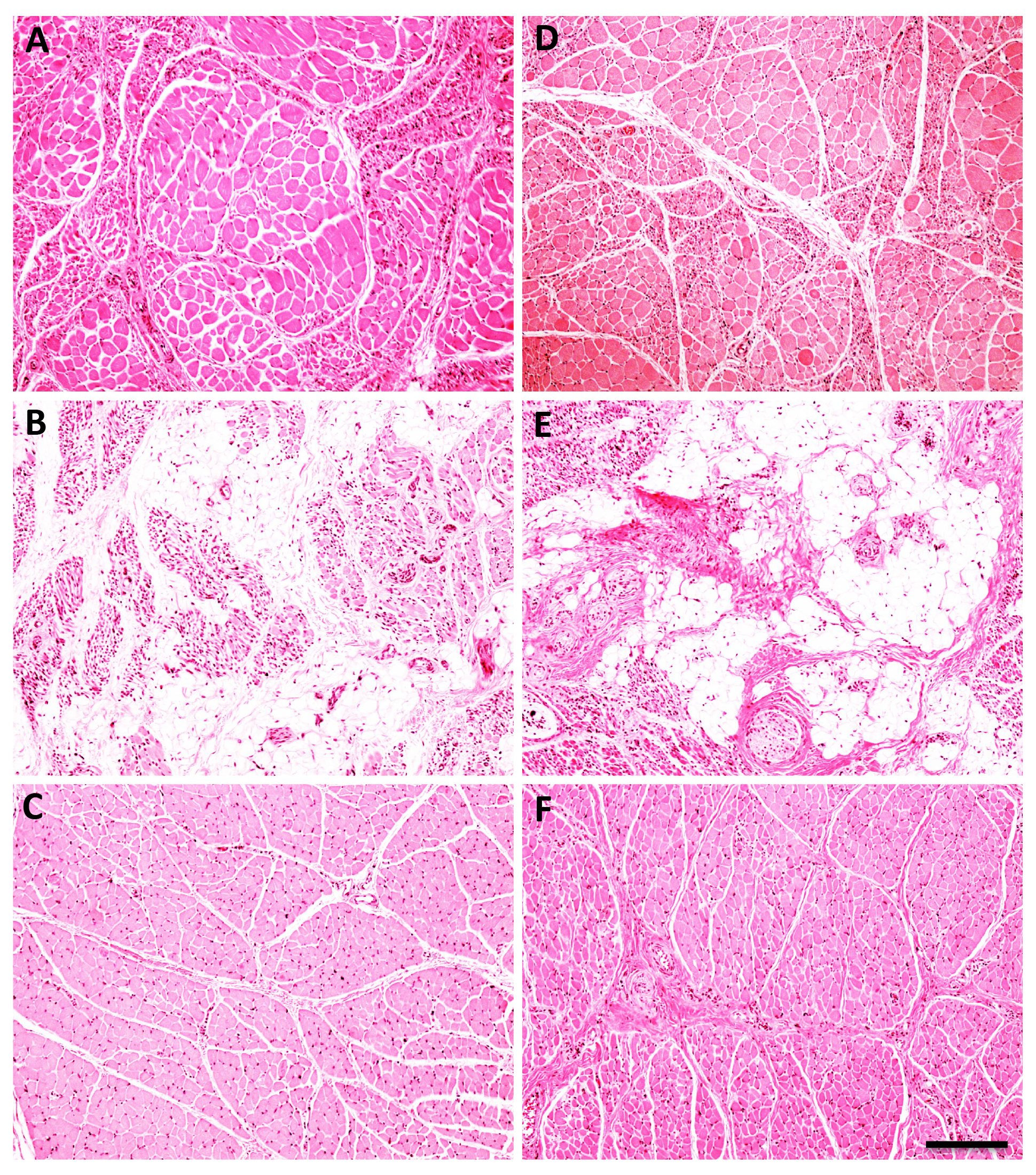

Supplement: Supplementary_Figure_ddae190 [file supplementary_figure_ddae190.zip › Supplementary Figure 2.tif]

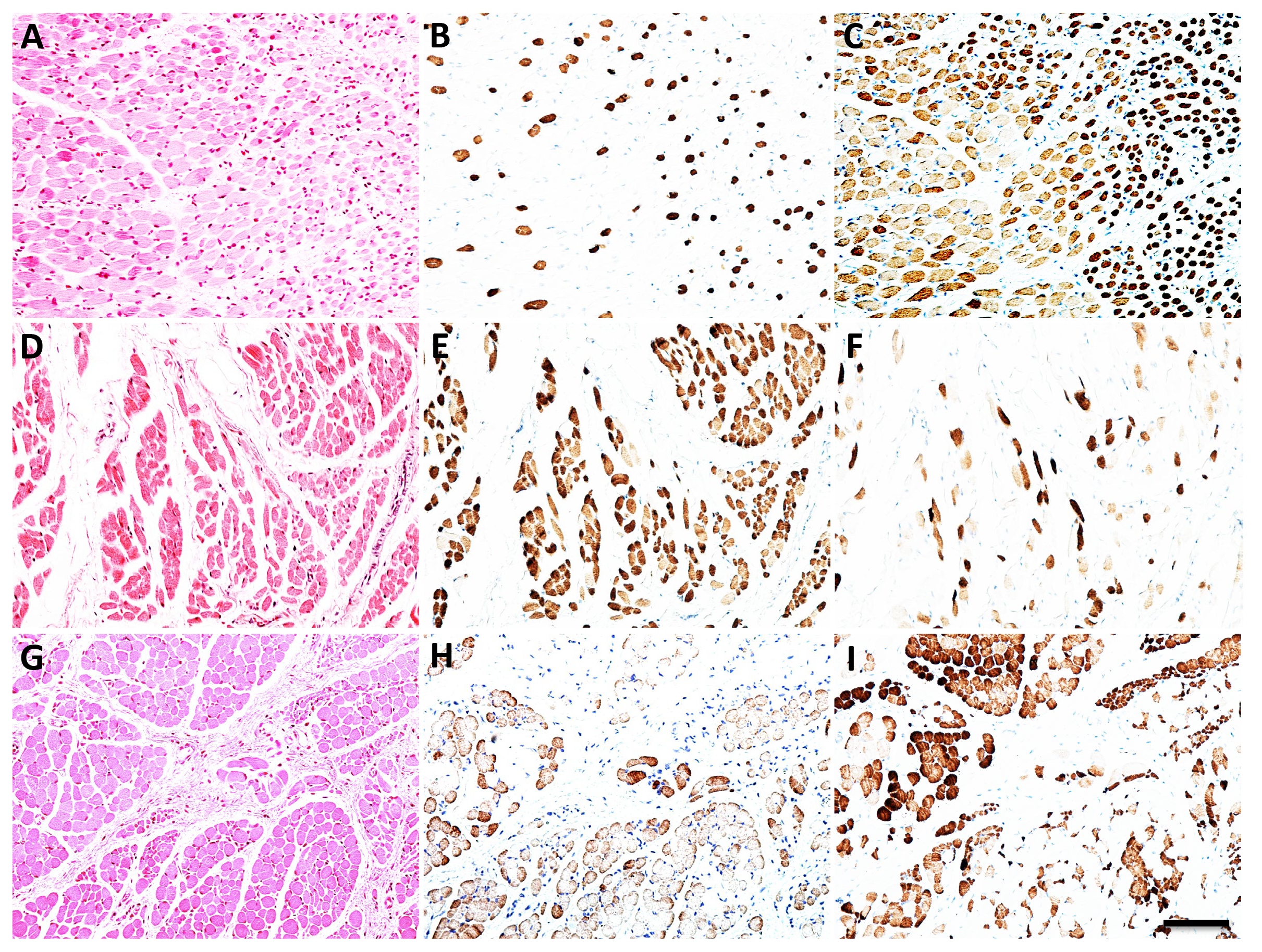

Supplement: Supplementary_Figure_ddae190 [file supplementary_figure_ddae190.zip › Supplementary Figure 3.tif]

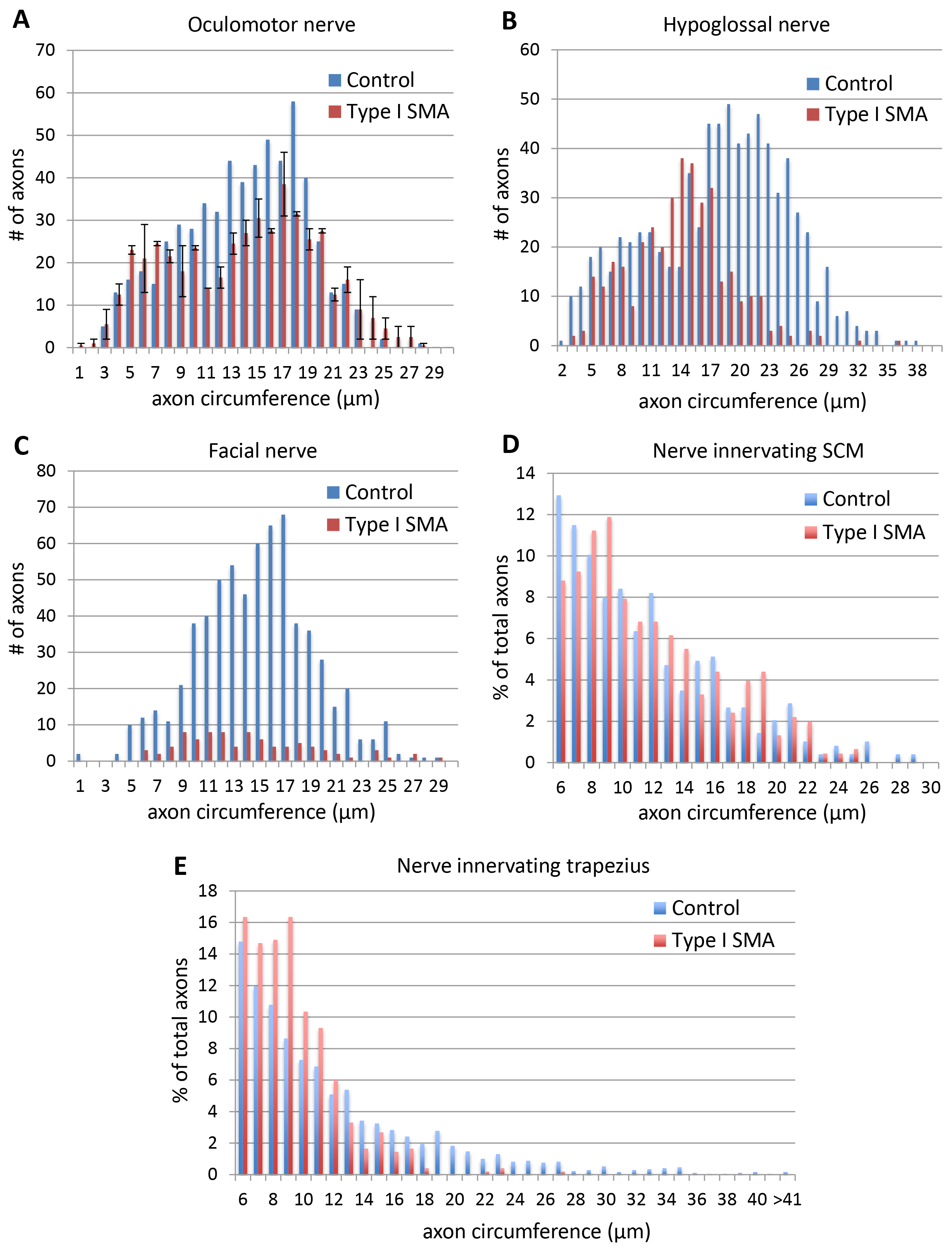

Supplement: Supplementary_Figure_ddae190 [file supplementary_figure_ddae190.zip › Supplementary Figure 4.tif]

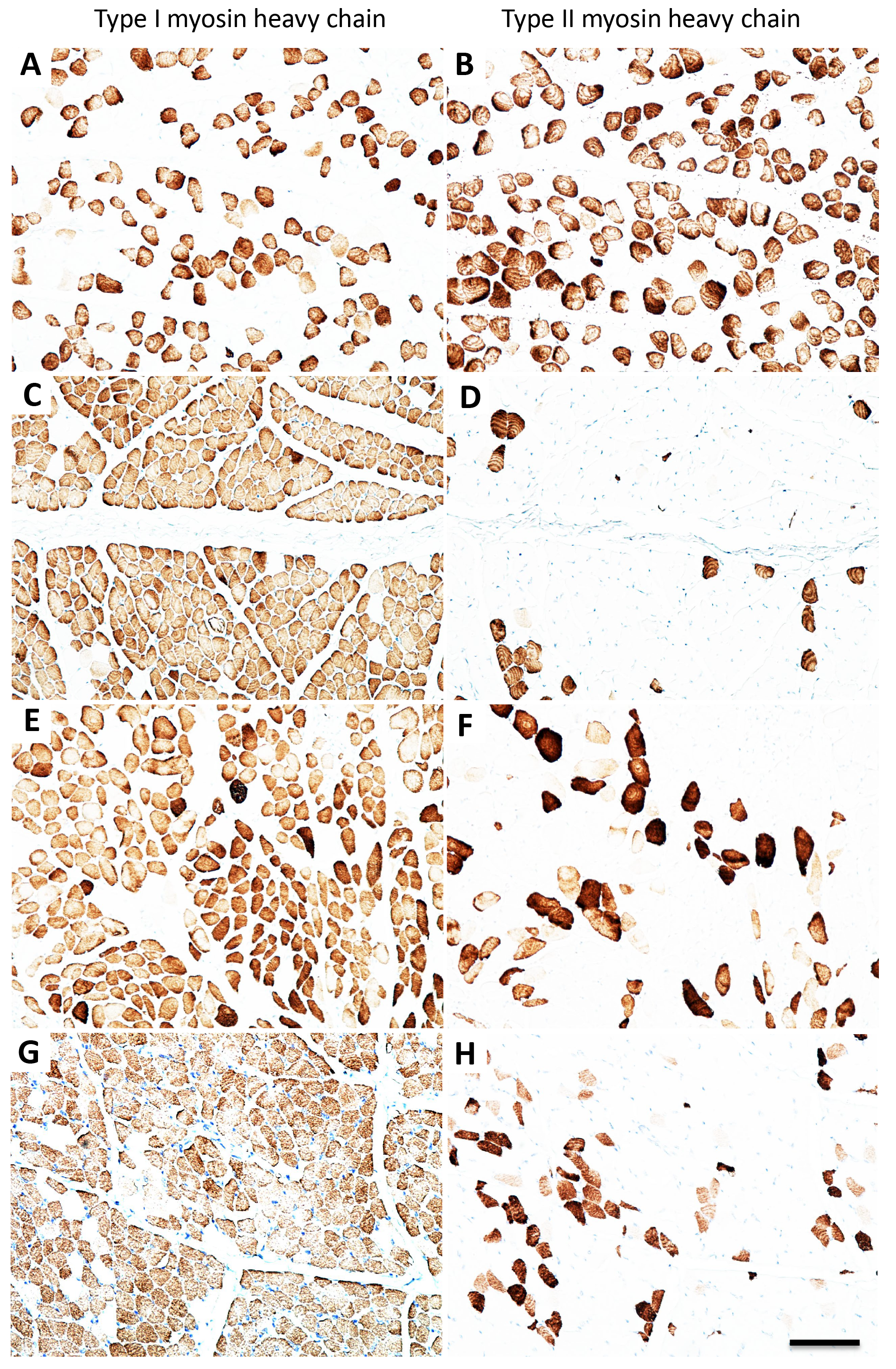

Supplement: Supplementary_Figure_ddae190 [file supplementary_figure_ddae190.zip › Supplementary Figure 5.tif]
