# Supplementary material for: Motor pool selectivity of neuromuscular degeneration in type I spinal muscular atrophy is conserved between human and mouse
Source: Hum Mol Genet. 2024 Dec 18;34(4):347–67. doi: 10.1093/hmg/ddae190 (PMC11811418; doi:10.1093/hmg/ddae190)
Supplement: Supplementary_Table_1_ddae190 [file supplementary_table_1_ddae190.docx]

**Supplementary Table 1**

| **Patient ID** | **Gender** | **Primary diagnosis** | **Age of diagnosis** | **Copies of SMN2** | **Respiratory support** | **Nutritional support** | **Cause of death** | **Week of birth** | **Weight at birth** | **Weight at death** | **Head circumference** | **Brain weight** | **Spinal cord length** | **Age at death** |
| --- | --- | --- | --- | --- | --- | --- | --- | --- | --- | --- | --- | --- | --- | --- |
| SMA-1 | Male | Type I SMA | 2 m.o. | N/A | No mechanical support | G-tube placed in 6th month of life | Respiratory failure/ pneumonia | 41 | 3350 g | N/A | N/A | N/A | N/A | 4 m.o. |
| SMA-2 | Male | Type I SMA | 4 m.o. | 2 | Night BiPAP at 6 m.o., full-time BiPAP at 8 m.o. | , | Respiratory failure/ pneumonia | Full-term | N/A | 7235 g | 46.0 cm | 965 g | 17.2 cm | 8 m.o. |
| SMA-3 | N/A | Type I SMA | N/A | N/A | N/A | N/A | Respiratory failure/ pneumonia | N/A | N/A | N/A | N/A | N/A | N/A | 8 m.o. |
| SMA-4 | Male | Type I SMA | 1 m.o. | N/A | Mechanical ventilation-dependent at 5 m.o. | G-tube placed in 4th month of life | Respiratory failure/ pneumonia | Full-term | N/A | 5700 g | 40.0 cm | N/A | N/A | 5 m.o. |
| SMA-5 | Male | Type I SMA | N/A | N/A | N/A | N/A | N/A | N/A | N/A | N/A | N/A | N/A | N/A | 7 m.o. |
| SMA-6 | Male | Type I SMA | N/A | N/A | N/A | N/A | N/A | N/A | N/A | N/A | N/A | N/A | N/A | 7 m.o. |
| SMA-7 | N/A | Type I SMA | N/A | N/A | N/A | N/A | N/A | N/A | N/A | N/A | N/A | N/A | N/A | 5 m.o. |
| SMA-8 | N/A | Type I SMA | N/A | N/A | N/A | N/A | N/A | N/A | N/A | N/A | N/A | N/A | N/A | 7 m.o. |
| SMA-9 | N/A | Type I SMA | N/A | N/A | N/A | N/A | N/A | N/A | N/A | N/A | N/A | N/A | N/A | N/A |
| SMA-10 | N/A | Type I SMA | N/A | N/A | N/A | N/A | N/A | N/A | N/A | N/A | N/A | N/A | N/A | N/A |
| SMA-11 | N/A | Type I SMA | N/A | N/A | N/A | N/A | Respiratory failure | N/A | N/A | N/A | N/A | N/A | N/A | 17 y.o. |
| Control-1 | Female | Congenital diaphragmatic hernia (CDH) | Prenatal | N/A | Intubated three times (months 1,6,7) | G-tube placed in 5th month of life | Respiratory failure secondary to CDH | 36 | N/A | 5630 g | 38.0 cm | 670 g | 18.6 cm | 7 m.o. |
| Control-2 |  | Ullrich Betham myopathy | N/A | N/A | N/A | N/A | N/A | N/A | N/A | N/A | N/A | N/A | N/A | 16 y.o. |
